# Cost-effectiveness of continuity of midwifery care for women with complex pregnancy: a structured review of the literature

**DOI:** 10.1186/s13561-018-0217-3

**Published:** 2018-12-05

**Authors:** Roslyn E. Donnellan-Fernandez, Debra K. Creedy, Emily J. Callander

**Affiliations:** 1 Transforming Maternity Care Collaborative, Nursing and Midwifery, Griffith University, Logan campus, University Drive, Meadowbrook, Queensland 4131 Australia; 2 Menzies Health Institute Queensland, Griffith University, Gold Coast, Queensland 4222 Australia

**Keywords:** Cost effectiveness, Midwifery care, Complex pregnancy, Continuity of midwifery care, Maternity models, Models of care, Health equity, Structured review

## Abstract

**Background:**

Critical evaluation of the cost-effectiveness and clinical effectiveness of continuity of midwifery care models for women experiencing complex pregnancy is an important consideration in the review and reform of maternity services. Most studies either focus on women who experience healthy pregnancy or mixed risk samples. These results may not be generalised across the childbearing continuum to women with risk factors. This review critically evaluates studies that measure the cost of care for women with complex pregnancies, with a focus on method and quality.

**Aims / objectives:**

To critically appraise and summarise the evidence relating to the combined cost-effectiveness, resource use and clinical effectiveness of midwifery continuity models for women who experience complex pregnancies and their babies in developed countries.

**Design:**

Structured review of the literature utilising a matrix method to critique the methods and quality of studies.

**Method:**

A search of Medline, CINAHL, MIDIRS, DARE, EMBASE, OVID, PubMed, ProQuest, Informit, Science Direct, Cochrane Library, NHS Economic Evaluation Database (NHSEED) for the years 1994 – 2018 was conducted.

**Results:**

Nine articles met the inclusion criteria. The review identified four areas of economic evaluation that related to women who experienced complex pregnancy and continuity of midwifery care. (1) cost and clinical effectiveness comparisons between continuity of midwifery care versus obstetric-led units; (2) cost of continuity of midwifery care and/or team midwifery compared to Standard Care; (3) cost-effectiveness of continuity of midwifery care for Australian Aboriginal women versus standard care; (4) patterns of antenatal care for women of high obstetric risk and comparative provider cost.

Cost savings specific to women from high risk samples who received continuity of midwifery care compared with obstetric-led standard care was stated for only one study in the review. Kenny et al. 1994 identified cost savings of AUS $29 in the antenatal period for women who received the midwifery team model from a stratified sub-set of high-risk pregnant woman within a mixed risk sample of 446 women. One systematic review relevant to the UK context, Ryan et al. (2013), applied sensitivity analysis to include women of all risk categories. Where risk ratio for overall fetal/neonatal death was systematically varied based on the 95% confidence interval of 0.79 to 1.09 from pooled studies, the aggregate annual net monetary benefit for continuity of midwifery care ranged extremely widely from an estimated gain of £472 million to a loss of £202 million. Net health benefit ranged from an annual gain of 15 723 QALYs to a loss of 6 738 QALYs. All other studies in this review reported cost savings narratively or within mixed risk samples where risk stratification was not clearly stated or related to the midwifery team model only.

**Conclusions:**

Studies that measure the cost of continuity of midwifery care for women with complex pregnancy across the childbearing continuum are limited and apply inconsistent methods of economic evaluation. The cost and outcomes of implementing continuity of midwifery care for women with complex pregnancy is an important issue that requires further investigation. Robust cost-effectiveness evidence is essential to inform decision makers, to implement sustainable systems change in comparative maternity models for pregnant women at risk and to address health inequity.

## Introduction and background

Review and transformation of maternity service models have been on the policy agenda of the Australian Government for the past decade [1, 2]. An important policy goal is to expand women’s access to midwifery caseload continuity of care in both the public and private health sectors [3, 4]. Continuity of midwifery care is where a named midwife provides full antenatal, intrapartum and postnatal care for a woman. The midwife provides physical, emotional and social support, flexible individualised care and robust multi-agency liaison. This enhances high quality perinatal care for mother and baby and strong working relationships with professionals [5]. Currently, only a small proportion of women are able to access continuity of midwifery care during their pregnancy [6].

Internationally, and in Australia, strong clinical and cost evidence already exist to support systemic implementation of continuity of midwifery care for women with healthy pregnancies [7–10]. However, in Australia the number of women who experience complex pregnancy is increasing [11]. In this review, complex pregnancy is defined as identified risk factors that place mother and/or baby at increased risk for adverse events. These can include biomedical and/or psychosocial risks, as identified by the woman and her care provider. Risk factors can be present at the start of pregnancy or arise at any time during the course of childbearing [12] . Evidence also shows significant inequity, poorer outcomes and associated increased healthcare costs for women who experience complex pregnancy. Outcomes for these women and their babies may potentially improve by increasing public health access to continuity of midwifery care models [13–17].

No previous systematic reviews have focused on women with complex pregnancies. To date, most systematic reviews of midwifery care have been conducted in the United Kingdom (UK) for low risk pregnancies. These reviews provide strong evidence for clinical and cost effectiveness of continuity of midwifery care (including birthing centres and home birth), as compared to obstetric - led units, but discrete economic analysis of outcomes and cost for pregnant women with risk factors were not included [9, 18–23]. Further, maternity services in many countries are not organised in the same configuration as in the UK, where clear delineation between continuity of midwifery care and obstetric-led units are an established feature.

Econometric models that applied productivity / efficiency frontiers and standard international resource ingredient approaches to develop predictive cost models, or other methods, for example, Net Benefit, were similarly limited [24, 25]. The implications of these studies is considered in the discussion in relation to costs of care for women who experience high risk pregnancy alongside clinical health outcomes in the midwifery continuity of care studies considered in this review. The lack of rigorous economic evaluation of different models of maternity care for women at high risk of complications has been emphasised in an integrated review examining cost data in relation to care provided in birth centres and at home with midwives [18]. This remains the case and provides a strong justification for the present review given the current evidence that show increasing rates of pregnancy complication and multiple complex maternal co-morbidity in Australia and elsewhere.

Capacity to improve maternity services to women with complicated pregnancy continues to pose a major challenge for the Australian health system [26–28]. This is particularly critical in rural and regional areas of the country where service options are limited and outcomes are significantly poorer than they are for women and babies in metropolitan areas [11, 29–31]. Critical evaluation of integrated evidence on the cost-effectiveness, resource use and clinical effectiveness of continuity of midwifery care for women who experience complexity therefore is an important consideration in quality review of maternity care.

## Aims and objectives

The aim of this review is to critically appraise available literature and summarise the evidence related to cost, resource use, and clinical outcomes of care for women with complex pregnancies who received care in a continuity of midwifery care model compared with other maternity models.

This structured, integrated review will examine the available evidence for cost-effectiveness of antenatal, intrapartum and postnatal care in continuity of midwifery care. It will critically evaluate the methods of these studies. This includes their capacity to support public health policy through expanded implementation and access to continuity of midwifery care for women who experience complications of pregnancy and childbearing and their babies.

## Method

This review used a stepped structured approach to documenting the search strategy [32]. The Matrix Method was then applied to ensure a systematic framework for article collection, organisation and analysis [33, 34]. Application of PRISMA guidelines strengthened credibility and transparency of the reporting and assessment process [35]. Use of eight quality appraisal questions from the recommended checklist for appraising the costs and benefits of economic evaluation studies enabled robust synthesis of the results of studies [36].

## Search

**Fig. 1:**
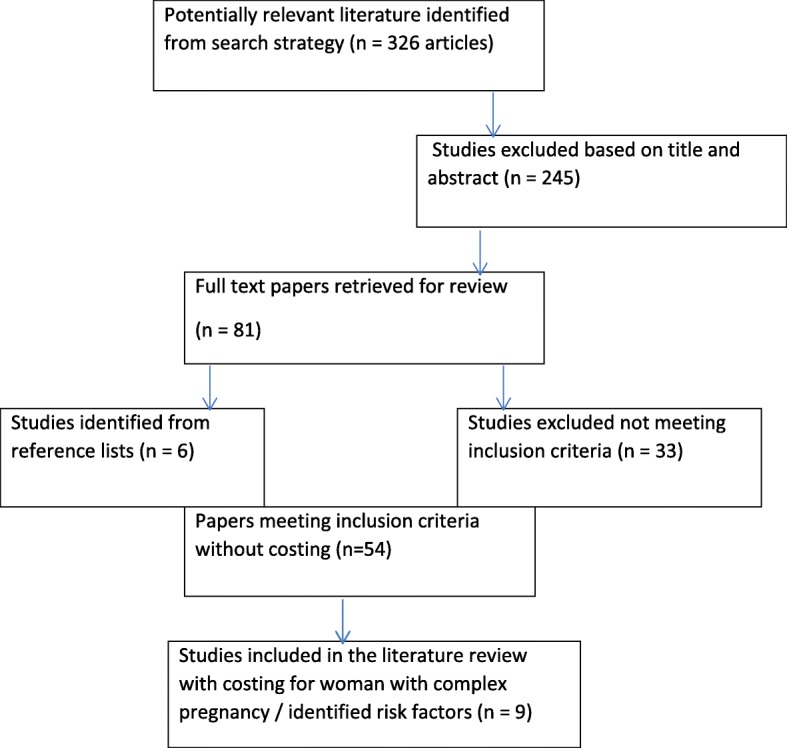
Flow Chart of study inclusion

Table 1 and Fig. 1 provide a summary of search details.

**Table 1:** Databases searched

Databases	
Medline, CINAHL, MIDIRS, DARE, EMBASE, OVID, PubMed, ProQuest, Informit Science Direct, Cochrane Library, NHSEED	
Published between 1994 and 2018	
English language publications only	
Article contained key search words or combined search terms: midwifery, midwife-led units, nurse-midwifery, birth centers, cost, cost-effectiveness, economic evaluation, economic outcomes, pregnancy risk classification, maternal outcomes, neonatal outcomes, clinical outcomes, maternity services	
Primary research article or Systematic Review/Meta-analysis or Integrative Review	
Economic analysis secondary to RCT accepted	
Peer-Reviewed Journals	
Population sample of childbearing women and/or their babies where risk classification profile defined and/or includes woman with high risk or complex pregnancy	
Measurement of at least one economic outcome measure combined with clinical and/or other outcome measures, in midwifery care units or integrated midwifery continuity models that included antenatal, birthing and postnatal services, compared to other maternity service models	
Economic perspective is funder/health service

## Inclusion criteria

Inclusion criteria were primary research articles published in English language, peer-reviewed journals between the years 1994–2018. The 24-year time - frame marked the emergence of studies on the cost-effectiveness of continuity of midwifery care, including the first Australian studies [10, 37, 38]. Non-English language papers were excluded, as were those that focused exclusively on low resource countries.

## Results

The classification of included studies within the evidence hierarchy is documented in Table 2.

**Table 2:** Summary of Included Studies

Evidence hierarchy level	Included studies
Level I: Systematic Review	Devane et al., 2010, Ryan et al., 2013, Sandall et al., 2016^a^
Level II Randomised Controlled Trial with Economic Evaluation	Homer et al., 2001a,b, Kenny et al., 1994, Rowley et al., 1995, Tracy et al., 2013
Levels III and IV Quasi-experimental Cost Studies (cohort, cross-sectional, case control, non-randomised prospective, retrospective audit)	Gao et al., 2014, Jan et al., 2004
Econometric Studies – predictive cost, productivity, resource models using datasets	No studies relevant to complex needs

^a^6 of 15 studies included in Sandall et al. 2016 review included cost/economic analyses – 4 of these cost studies included woman of mixed risk and were included in this review

## Appraisal of studies

This review identified three systematic reviews that examined the cost and clinical effectiveness of continuity of midwifery care and obstetric-led maternity models. All three of these reviews were undertaken in the UK (Table 3).

**Table 3:** Summary of Three Systematic Reviews^a^

Aim of Study	Sample / Setting	Design	Major Cost Findings	Health Outcomes	Strengths / Limitations
1. Sandall et al. (2016) Compare effects of midwife-led continuity models with other models for childbearing women and their infants Primary outcomes antenatal, birth & immediate postpartum outcomes Secondary outcomes birth intervention, morbidity, some aspects of resource use & cost United Kingdom √	Included: 15 RCTs 17 674 women (Canada, Ireland, Australia, UK) Excluded: 22 studies Only 6 of the 15 RCTs measured cost of model; only 4 of the 6 RCTs that measured costs included “mixed risk” pregnant women/high risk pregnancy: Kenny 1994 Rowley 1995 Homer 2001 Tracy 2013	Systematic review Cochrane Pregnancy & Childbirth Group Trials Register + reference lists of retrieved articles. Selection criteria: published and unpublished trials, pregnant women randomly allocated to midwife-led continuity models of care or other models of care for pregnancy & birth Cost trend reported narratively as RCT cost method varied, e.g. cost analysis; CEA; or not stated	Trend to cost saving effect in midwife-led continuity Cost savings intrapartum care – all studies Antenatal: varied Postnatal: 1 study higher cost/ 1 study no difference Primary 0utcome in midwife – led models (RR) (CI) ^↓^ regional analgesia (0.85, 0.78 – 0.92) ^↓^instrumental birth (0.90, 0.83 – 0.97) ^↓^ pre-term <37 wk (0.76, 0.64 – 0.91) ^↓^ fetal loss <24 wk (0.84, 0.71 – 0.99) ^↑^spontaneous vaginal birth (1.05, 1.03 – 1.07) No difference CS or intact perineum Secondary 0utcome midwife – led models: ^↓^amniotomy; ^↓^ episiotomy; ^↓^ fetal loss <24 wks; No labour analgesia; longer labour (MD) 0.50 hrs, No difference for: fetal loss >24 wks; labour induction; A/N admission; A/N haemorrhage; augment labour; PPH; low birthweight; 5 min Apgar < 7; SCBU admission; initiate breastfeeding	Time horizon: RCT (cost included) 1994 – 2013 Women receiving midwife care less likely to have epidural, episiotomies, instrumental birth. Spontaneous vaginal birth rate increased. CS rate no difference. Women less likely to have pre-term birth, lower risk of losing babies < 24wks, More likely to be cared for in labour by a known midwife. No adverse effects compared with other models. Conclusion: most women should be offered midwife-led continuity of care BUT Evidence may not apply to women with serious pregnancy or health complications as these women were not specifically included in all studies / analysis for clinical effectiveness not stratified	Limited evidence CEA for women with complex pregnancy Combined results: low and mixed risk pregnant women 4 studies used different economic evaluation methods -: narrative report as cost assessment inconsistent Strong evidence cost improved in midwifery models for low risk with, reduced intervention + increased satisfaction. Mixed risk studies - ‘interpret with caution’
2. Ryan et al. (2013) Analysis of evidence on cost – effectiveness of midwife-led care compared with consultant –led care in UK settings. Estimate potential cost savings to accrue from expansion of midwife – led care in UK Used Section 3 CE of Devane et al. 2010 SR United Kingdom √	Economic synthesis of 3 RCTs evaluated against guidelines for economic review Drummond and Jefferson (1996) 5796 women Hundley 1995 Young 1997 Begley 2009 Excluded: Flint 1989 (sub-group costing 49 of 1001 women only)	Systematic review 12 electronic databases for cost midwife led models: Cochrane Methodology Register NICE methods + multiple 1-way sensitivity analysis for economic synthesis of costs used 3 RCTs applied to 8 scenarios CE measure used Incremental Net Benefit (INB): expressed as Net Monetary Benefit (NMB) – £ value, and Net Health Benefit (NHB) – QALY, Quality adjusted life year gain	Mean cost saving £12.38 per woman midwife led (ML) care Expansion of ML care to 50% of all eligible women in UK projected aggregate £1.16 mil cost saving/yr Sensitivity analysis: cost change per woman varied from saving £253.38 (37.5 QALYs gained per year) to cost increase £108.12 dependent on assumptions with correspondent aggregate annual savings £23.75 million, or aggregate annual cost increase £10.13 million	Time horizon: RCT (cost included) 1995 – 2009 Three economic analyses used in synthesis of potential cost saving from increasing midwife-led services for eligible maternities. Issues identified around generalizability of findings. High rate of transfer from ML to medical-led care in studies demonstrates ‘risk’ assessment criteria unable to identify all women who will develop complications in pregnancy and labour	Rigorous health economic assessment measures: INB, NMB,QALYs Limited to UK system Excluded RCTs from Australia and other countries where no comparison with consultant-led model Mixed risk pregnancy profile; sub-group analysis show cost results consistent for groups as (RR) fetal loss and neonatal death overlap with 1.00
3. Devane, D. et al. (2010). Section 3: assessed CE of midwife-led care compared with consultant –led care. Estimated potential cost savings of expanding midwife-led care in UK (pp. 33–45) United Kingdom √	Based on 3 of 4 RCTs See 2. above Hundley 1995 2844 women; Young 1997 1299 women; Begley 2009 1653 women	Systematic review see 2. above Sensitivity analysis x 3 based on 8 scenario SA 1: Systematically varying estimated cost savings SA 2: Systematically varying RR for overall fetal loss & neonatal death using low risk and ‘mixed risk’ cases SA 3: Systematically varying assumed uptake of ML service	As published in Ryan, Revill et al. 2013	Time horizon: RCT (cost included) 1995 – 2009 Expanding midwife –led maternities show: Reduced rate of interventions in ML continuity of care, including: <AN hospitalization Reduced use of regional analgesia in birth, less episiotomy and instrumental delivery & greater numbers of women more likely to experience spontaneous vaginal birth BUT may not extrapolate to women with identified risk factors	Cochrane bias assessment tool used for trial internal validity Not generalisable, small number of studies CE varied with unit size, location and volume

^a^Articles presented in reverse chronologic order; **√** denotes a minimum PRISMA score of 20 based across a possible total of 27 check-list items

A summary of the six primary studies included in this review, including study design is provided in Table 4. All studies were completed in Australia.

**Table 4:** Primary Articles Reviewed^a^ Study design

Aim of Study	Sample / Setting	Design / Method	Model used (link costs & health outcomes)
1. Gao, Y. et al. (2014). Compared CE two models, Midwifery Group Practice (MGP) against baseline cohort of Aboriginal mothers / infants. Clinical and cost analysis Australia **√**	Regional hospital, Northern Territory MGP cohort: 7 communities MGP Women = 310 MGP Babies n = 315 (Sept 2009 – June 2011) Baseline cohort: 2 communities Baseline Women n = 412 Baseline babies n = 416 (Jan 2004- Dec 2006) All risk	Economic evaluation - retrospective records audit (Baseline Jan 2004-Dec2006) prospective data collection (MGP Sept 2009-June 2011)	Cost-consequences analysis: Australian dollars Measured/calculated direct costs per group Established comparative cost and changes post establishment MGP service from first antenatal appointment to 6 weeks postpartum for Aboriginal mothers and babies
2. Tracy, S.K. et al. (2013). Assess efficacy, safety and cost of caseload midwifery versus standard hospital maternity care for women of mixed obstetric risk Dec 2008 -May 2011 Australia √	Women of all pregnancy risk status (not stratified) Sample 1748 women 2 tertiary teaching hospital sites, 2 states, NSW / Queensland	2 arm RCT Caseload care, Women with a named midwife *n* = 871 versus Women Standard Hospital Care *n* = 877 Intention to treat analyses	Cost- consequences analysis: Australian dollars Cost of care per woman based on DRG separation and direct and indirect costs for resource use collected from hospital financial system Primary & secondary clinical & cost outcomes Univariate logistic regression, OR 95% CIs and Pearson χ2 test; p values; non-parametric bootstrap percentile CIs infer significance of effects
3. Jan S. et al. (2004). Holistic economic evaluation of an Aboriginal Community Controlled Midwifery Program in Western Sydney 1990-1996 Australia **√**	Sample: 2 groups of Aboriginal women, Western Sydney birthing between Oct 1990 – Dec 1996, Nepean & Blacktown hospitals *n* = 834 Antenatal care at Daruk Aboriginal Community Controlled Program, or either hospital	Cost analyses estimated Direct Program costs and downstream savings. Retrospective case record audit	Cost analysis: Australian dollars Clinical and cost data linked from case record and NSW Midwives Data Collection 1991–1996 with hospital data linked with Australian National DRG cost weights; Medication: PBS (pharmaceutical benefits) Diagnostic tests: MBS (medicare benefits) Sensitivity analysis used to model uncertainty
4. Homer C.S. et al. (2001). Assess clinical and cost difference – team community midwifery care -CMWC compared to control/ standard hospital care - SHC 1997-1998 Australia **√**	Sample of women of mixed pregnancy risk *n* = 1089 CMW = 550 SHC = 539 One Australian public hospital State of NSW	RCT-Zelen Design Cost analysis: CMW vs SHC 2 teams each with 6 fulltime midwives provided care for 600 women/yr (25 births/mth/team) Calculated mean cost/woman for 9 components of maternity care	Cost analysis: Australian dollars Mean cost/woman/group - standard errors and 95% CI calculated using bootstrap technique Components of care and cost for resources used for each woman: antenatal clinic; antenatal admission; day assessment unit; labour and birth; hospital-based postnatal care; domiciliary postnatal care; and, admission of neonates to the special care nursery (SCN), on-call costs. Salaries and wages calculated at market prices Sensitivity analysis in 3 areas: Neonatal admission to SCN; Efficiency of AN clinics; Proportion of elective CS
5. Rowley, M.J. et al. (1995). Examined cost/clinical differences for birth between 2 groups - Team Midwifery - 6 midwives vs routine hospital care Australia **√**	Sample of women of mixed pregnancy risk *n* = 814 Discrete stratification of high risk = 275 women Team midwifery *n*= 405 Hospital care n = 409 One Australian public hospital State of NSW	RCT: 2 groups continuity team (midwives) vs routine care (hospital) Cost measured: Australian National Cost Weights for Diagnostic Related Groups (DRG) per birth / delivery Intention to treat	Cost-effectiveness: Australian dollars; direct costs Multiple outcomes measured. No single measure of effectiveness derived. Australian national cost weights for diagnosis-related groups (DRGs) applied to outcomes of women for whom complete results were avail. Performed retrospectively by clerk blinded to study - based on medical records, covered inpatient costs. Cost of intervention & comparative care estimated by analysing midwives' salaries. No discounting as time-period < one year. Costs and quantities not reported separately. No sensitivity analysis undertaken. No price dates given.
6. Kenny, P. et al. (1994). Cost analyses: Team Midwifery Vs Standard hospital care. Included clinical outcomes Sept 1992 – July 1993 Australia **√**	Sample n = 446 women Team Midwifery n =213 Standard Care n = 233 Westmead public hospital State of NSW	RCT 2 Arm Study Resource cost estimates: AN, birth, PN care Cost estimated where statistically significant difference in service use shown Included: direct costs, infrastructure, staff salaries - calculated for ‘low’ and ‘high’ risk women each group	Cost analysis (Drummond1987) Costs estimated based on resource use at AN, birth and PN (including domiciliary) stages of care separately Costs based on care delivered No sensitivity analysis undertaken. Costing assumptions: cost effective if resource costs of midwifery care shown to be less or equivalent to conventional care and health benefits of midwife care relative to conventional care are shown to be positive

^a^Studies are presented in reverse chronologic order; **√** denotes a minimum score of 6 (from possible 8) quality appraisal questions; Studies 2, 4, 5 and 6 = randomised controlled trial with linked economic evaluation

Results of these primary studies are reported in table 5.

**Table 5:** Primary Articles Reviewed^a^ Study results

Study	Major Cost Findings	Health Outcomes	Strengths / Limitations
1. Gao, Y. et al. (2014)	Cost saving AUS $ 703 / mother-infant episode for MGP cohort was not statistically significant (*p*=0.566) MGP (midwifery model): ^↓^birth cost -$ 411, *p*=0.049 ^↓^SCN cost – $ 1 767, *p*=0.144 ↑ AN cost + $ 272, *p*<0.001 ↑PN cost + $ 277, *p*<0.001 ↑infant readmission costs + $ 476, p=0.05 ↑travel cost = $ 115, *p*=0.001	Time horizon: Midwife cohort – all Aboriginal mothers who gave birth between Sept 2009 - June 2011 (and their infants) Baseline cohort – all Aboriginal mothers who gave birth between Jan 2004 – Dec 2006 (and their infants) Women who received midwife model had more antenatal care, more ultrasounds, were more likely to be admitted to hospital in antenatal period, had equivalent birth outcomes (i.e. mode of birth; pre-term birth; low birth weight) compared with baseline cohort. Babies in midwife model admitted to Special Care Nursery had significantly reduced length of stay	Mixed risk; small sample Cost assumptions used for economic analysis – expert opinion not primary data Missing data (3.7% – 24.5%); 51% all cases = missing data; Time trend confounding; Hostel costs & transport costs not included
2**.** Tracy, S.K. et al. (2013).	Median cost saving of $ 566 AUS / woman with Caseload / named midwife	Time horizon: Dec 2008 – May 2011 Birth interventions reduced in midwifery model 30% > spontaneous onset of labor; ↓ analgesia; ↓elective caesarean; No significant difference for overall rate of caesarean between groups. Similar safe outcomes for mothers and babies between groups	Registered Trial: ACTRN12609000349246 All pregnancy risk status No stratification of risk profile Defined eligibility, inclusion/exclusion criteria Study sufficiently powered (80%) and Type 1 error 5% Sample bias challenged external validity Cross-overs – did not receive assigned model of care Non-masking of group allocation from clinicians
3. Jan S. et al. (2004).	Net cost estimate AUS$1, 200 per client – calculated by subtracting cost savings to other centers Daruk Antenatal service saw 245 women for 339 pregnancies during study	Time horizon: Women birthing between Oct 1990 – Dec 1996 No significant difference in service birth weights or perinatal survival Daruk Antenatal care = Gestational age @ 1’st visit lower; mean number AN visits higher; attendance for AN tests better Women strongly positive toward midwife model for relationship, trust, accessibility, flexibility, information, empowerment and family-centered care	Mixed risk pregnancy Evaluation framework, both quant and qual methods Focused on antenatal care attendance and access; costs were broader than used in conventional economic analyses - included birth outcomes and antenatal attendance in a subsequent pregnancy Assumptions in sensitivity analyses / estimated downstream health costs
4. Homer C.S. et al. (2001).	Mean cost/woman: CMWC A$2 579 vs SHC A$3 483 Excluding neonatal costs: CMWC A$1 504 (1449–1559; 95%CI) v SHC A$1 643 (1563–1729 95%CI) Mean cost saving 9 areas SHC – CMCW: Antenatal +28.84 Day Assessment Unit -5.42 Antenatal inpatient +38.74 On-call cost -21.81 Labour / birth +68.83 Hospital Postnatal care 43.85 Domiciliary care -11.06 Special Care Nursery +2801.28 Total/woman +904.09	Time horizon: 1997 – 1998 (not specific) Caesarean rate: CMWM 13.3% vs SHC 17.8% (OR . 0.6, 95% CI 0.4±0.9, P = 0.02) No other significant differences were detected among women or babies for clinical outcomes or events during labour and birth between care models	Cost analysis alongside RCT; 10 000 bootstrap replications Mixed risk sample; Costs included resource use, clinician travel, neonate care; No equipment, capital or program development costs; No transfer rates; Caseload/midwife key to cost saving; Not possible to determine optimal caseload numbers; unclear if data analyzed by intention to treat
5. Rowley, M.J. et al. (1995).	Mean cost ↓4.5% per birth: Team MW v Routine care A $3 324 vs A $3 475	Time horizon: May 1991 – June 1992 Included first AN visit to 6 weeks after birth Team MW women: higher AN class attendance OR 1.73; 95% CI:1.23-2.42 ↓ birth interventions 36% vs 24% OR; 1.73 (1.28 – 2.34); p<0.001 ↓ pethidine use 0.32 (0.22 – 0.46) ↓ newborn resuscitation 0.59 (0.41 – 0.86) Maternal satisfaction with team care was greater on 3 elements: information giving; participation in decision-making, and relationships with caregivers. Less cost than routine care and fewer adverse maternal and neonatal outcomes	Cost study alongside RCT Included women of all pregnancy risk status Model was team midwifery care, not caseload continuity Costs based only on DRGs; i.e. top – down cost only / not detailed. Unable to compare with other economic evaluations
6. Kenny, P. et al. (1994).	Team Midwifery vs Standard Care: Avg costs AN cost/woman High risk $ 427 vs $ 456 Low risk $ 135 vs $ 133 Average additional cost per birth / woman $ 4.21 vs $ 9.36 PN cost/woman: Hospital stay $ 356.64 vs $ 397.26 (earlier discharge) Domiciliary $45.45 vs $45.80	Time horizon: Sept 1992 – July 1993 Significant differences: manipulative vaginal birth, episiotomy & perineal tears. Women in team midwife care reported higher levels of satisfaction over 3 periods of antenatal, birth and postnatal care with information, communication and midwife attitude and skill	RCT Level 1 evidence; All risk pregnancy included; Discrete costs: AN, birth and PN Robust, bottom-up costing; Team midwife model, not caseload; Low risk of bias, although blinding not stated; Loss to follow up - 19 in TM vs 22 in SHC

^a^Studies are presented in reverse chronologic order; **√** denotes a minimum score of 6 (from possible 8) quality appraisal questions; Studies 2, 4, 5 and 6 = randomised controlled trial with linked economic evaluation

## Quality of studies

Economic evaluations undertaken alongside randomised controlled trials (RCT) constituted the most robust evidence for economic analysis of continuity of midwifery care models available. Importantly, four RCTs included in the systematic review by Sandall et al. [9] were conducted in Australia and included women of mixed pregnancy risk classification. The four Australian RCTs, were the only studies that examined cost results for continuity of midwifery care models that also included women with identified pregnancy risk factors [10, 37–39]. Two of the RCT studies identified their economic evaluations as cost analyses, Homer et al. [39], Kenny et al. [37]. One other was identified as a cost-effectiveness study on the NHS EED data base (Rowley et al. [38] and the remaining cost consequences analyses study, Tracy et al. [10], calculated per woman cost of care based on DRGs as well as direct and indirect costs for resource use.

The quality of cost, resource use and clinical effectiveness evidence in the primary studies included in this review therefore is high as they include mainly RCT evidence and also incorporated results from Levels III & IV of the evidence hierarchy. However, of the four RCT studies, three involved team midwifery models, as contrasted with continuity of care with a named midwife. In a team midwifery model a small group of midwives (up to 6 and no more than 8) provide care for identified women and the degree of continuity is not as high as in continuity of care with a named midwife. Other study designs considered cost evidence with varying levels of quality. Based on the NHMRC evidence hierarchy, the studies, in order of decreasing quality included cost analysis [40], and cost consequences analyses based on retrospective records audit [41].

While a number of studies were identified that used internationally validated ratios to model predictive costs for mode of birth and other interventions, these studies were excluded as they were not directly applied to continuity of midwifery care or women with identified pregnancy risk factors [42–45].

Four areas of economic evaluation that relate to women who experienced complex pregnancy were identified from the review:

1. *Comparisons of midwife-led versus obstetric consultant-led units for cost and clinical effectiveness*

Two studies had data relevant to this theme. While cost models were based on trials that recruited women with low pregnancy risk, sensitivity analysis modelled cost for women of mixed pregnancy risk classification based on UK population data. In the two systematic reviews from the UK where continuity of midwifery care and obstetric consultant-led maternity models are common, an estimated mean cost saving for each eligible woman of £12.38 was found in the continuity of midwifery care model overall. This provided aggregate health savings of £1.16 million per year for the health system if only half of all eligible women received continuity of midwifery care [20]. However, it is of note that these results are highly sensitive to assumptions, particularly changes in the rate of fetal loss and neonatal death, as well as the midwife’s caseload. When sensitivity analysis was applied to include women of all risk categories and the risk ratio for overall fetal/neonatal death was systematically varied based on the 95% confidence interval of 0.79 to 1.09 from pooled studies, the aggregate annual net monetary benefit for continuity of midwifery care ranged extremely widely. This varied from an estimated gain of £472 million to a loss of £202 million. Net health benefit ranged from an annual gain of 15 723 QALYs to a loss of 6 738 QALYs. Additionally the midwife’s caseload needs to be sufficiently large to attain operational efficiencies, otherwise the cost per maternity increases. The conclusion therefore is that the evidence base for cost-effectiveness of continuity of midwifery care for women with pregnancy risk is limited [19, 20]. As stated, these findings are limited to the UK context where midwifery-led and obstetric-led units are an established feature of the health system. This is not the case in other contexts, including Australia.

2. *Cost of continuity of midwifery care and/or team midwifery compared to Standard Care (medical)*

Over the past two decades economic evaluations conducted alongside RCTs in several Australian states have demonstrated cost saving and clinical effectiveness of continuity of midwifery care models compared with standard hospital care in the same setting [10, 37–39, 46]. Some of these studies focused on ‘team midwifery’ [37–39, 46] while others evaluated continuity of midwifery care models. In a ‘team model’ there is no primary care provider and the level of continuity is variable, compared to continuity of midwifery care models where named midwives provide services for women across the full continuum of antenatal, birth and postnatal care [9]. The cost evaluations of team midwifery and one cost evaluation of continuity of midwifery care in Australia have included pregnant women of mixed-risk status [10]. However, all these studies, with the exception of Kenny et al. [37] did not stratify results specific to women with high-risk pregnancy.

The most recent mixed-risk Australian trial identified a median cost saving of A$566 for women who received continuity of midwifery care compared to standard hospital care services, these savings cannot be generalised to high risk groups [10]. This trial identified safe outcomes for mothers and babies but no significant difference between continuity of midwifery care and standard care for primary outcomes of epidural analgesic use during labour, number of CS, instrumental vaginal births or unassisted vaginal births. Earlier rigorous cost analysis of community-based continuity of midwifery care model for all-risk women in Australia also identified mean cost savings per woman of A$804 in the continuity of midwifery care model. This included a significant difference in the rate of CS [39]. After neonatal costs were excluded in this study, mean cost savings continued to favour women and babies in the continuity of midwifery care model by A$139 [39]. While it was not possible to determine optimal service volume based on caseload numbers, the number of women booked for care in the continuity of midwifery care model was one of the important keys to cost-effectiveness. The reason for this relates to efficiency and savings generated by the volume of women able to be allocated to a maternity model in relation to the staff ratio required to provide maternity services [20, 47].

Earlier team midwifery RCT studies identified reduced levels of birth intervention in addition to modest cost savings for women of all-risk. One study identified as a cost-effectiveness study used Australian Diagnostic Related Groups ‘top-down costing’ that showed a mean cost reduction for birth of 4.5% for women in the midwifery group, [38]. The other study, a cost analysis, analysed discrete costs (‘bottom-up costing’) for each episode of service (i.e. antenatal, birth, and postnatal care) in the midwifery model versus standard hospital care [37]. Specific cost for high- and low-risk pregnancy episodes of care is shown in Table 4. Kenny et al. [37] is the only study identified that separated the risk stratification profile of women in their all-risk pregnancy sample in relation to costs. All the studies suggested a cost saving in intrapartum care in the midwifery model. One study suggested higher cost and one study showed no difference in cost of postnatal care in the midwifery model compared with the medical-led model. Cost results for postnatal care also were not stratified as specific to women with pregnancy risk.

3. *Cost-effectiveness of* continuity of *midwifery care for Aboriginal women versus standard care*

Two studies attempted to measure the cost of continuity of midwifery care in identified Australian populations with higher pregnancy risk status. Gao et al. [41] used a retrospective baseline cohort measured against a prospective cohort of pregnant Aboriginal women (all-risk status) to identify cost changes from the first antenatal visit through to six weeks postpartum after introduction of continuity of midwifery care. While there was a trend for cost savings of A$703 for women at 6 weeks, these were not significantly different from baseline costs. Limitations of the study included small sample size, cost assumptions (hostel and transport were not included), and missing data (51% of all cases). While no significant difference in major birth outcomes was identified antenatal attendance and hospital admissions increased, and average length of special care nursery stay for the babies of the women decreased.

An earlier cost analysis of a metropolitan, Aboriginal-controlled, continuity of midwifery care service (all-risk) estimated direct program costs and downstream savings in the health sector of A$1,200 per woman [40]. Downstream savings projected longer - term cost benefits that were gained, for example, from reductions in resource use experienced by associated services. The study used Australian National DRG cost weights [48] and cost data from Medicare and the Pharmaceutical Benefits Scheme [49] and sensitivity analysis to model uncertainty. Costs included were broader than those used in conventional economic analyses. Among the additional cost considerations were clinical outcomes for birth, antenatal attendance in a subsequent pregnancy, and subtraction of cost savings to other centres. While more recent clinical evaluation of midwifery models of care for Aboriginal women have demonstrated significant improvement in infant birthweight and perinatal survival, specific cost analysis of these benefits have not yet been undertaken as part of the studies [15, 50].

4. *Patterns of antenatal care for women of high obstetric risk and comparative provider costs*

In this review antenatal care provided by midwives for high risk and mixed risk samples showed reduced cost in three RCT studies [20, 37, 39] and increased cost in two others (non-RCT) [40, 41](as shown in Tables 3 and 4).

This is consistent with a cochrane review of patterns of antenatal care which showed, among different providers of antenatal care (midwife, general practitioner, obstetrician), primary outcome measures of low birthweight, pre-eclampsia/eclampsia, severe postpartum anaemia, and treated urinary tract infection (all high risk factors for pregnancy complications including pre-term birth) demonstrate similar clinical effectiveness [51, 52].

## Discussion

Increasingly health services need to justify quality outcomes as well as value for money [53–56]. Quality maternity care is especially important for women who experience high risk pregnancy as inequitable health outcomes for these mothers and babies pose additional policy and service implementation challenges for government [57, 58]. Moreover, decision-makers often grapple to determine the most effective and sustainable models of care to close these gaps [28, 59, 60]. In high resource settings such as Australia, many women with the most significant health inequities also experience pregnancy complications with long-term comorbidity [11, 30]. The public health burden, including the cost of chronic disease for these women, their babies and the health system is higher and often lifelong [61]. Economic evaluation to inform decision-making regarding the comparative cost-effectiveness of different maternity models across the continuum of childbearing therefore should be a high priority.

This review demonstrated that there are few studies specific to evaluating cost-effectiveness of midwifery continuity of care models for women who experience high-risk pregnancy relative to other models of maternity care, including standard and traditional obstetric led models. Of the studies included, significant limitations and caveats apply. Inter-country comparison of cost and models of maternity care between health systems that do not share the same features prohibit comparative generalisability of both outcomes and models of care, including the costs attributable to different models and systems. The costs and outcomes may vary widely according to structural factors such as funding model and workforce arrangements and the influence of demographic features and characteristics of woman who experience high - risk pregnancy within the study samples.

A strong international evidence base supports woman’s early access to antenatal care, pre-natal education and health promotion strategies provided by midwives as an effective intervention to improve maternal and neonatal outcomes when integrated with other specialised health and social support services [57, 58, 60]. Poor access to antenatal care, including delayed attendance for the first visit is associated with higher rates of pre-term birth and low birthweight infants and increased interventions in late pregnancy, all of which have been found to negatively impact cost [11, 62]. This review found a small limited evidence-base to support the delivery of cost -effective antenatal care by midwives to women with identified pregnancy risk factors that deliver equivalent and/or improved health outcomes for them and their babies when compared to standard or traditional models of obstetric care [10, 37–39]. Additionally, while earlier systematic review has shown that low-risk pregnant women who receive midwifery-led care require fewer antenatal visits, generating significant short-term cost savings for services [52], this is not always the case where women have identified medical and psychosocial risk factors.

Two studies included in this review identified higher antenatal costs associated with increased frequency of visits for women identified with higher pregnancy risks who may otherwise experience increased morbidity and mortality in pregnancy and childbearing [40, 41]. Consideration of overall ‘downstream’ savings’ within midwifery continuity of care models for women with risk factors therefore is a relevant consideration. It is recommended that future analyses include measures and methods broader than those used in conventional economic analyses, for example, longer term modelling of disutility costs associated with onset of chronic disease states [63]. Downstream savings have been demonstrated to be important in estimating both program and health sector costs accurately, particularly where access and significant health inequities have been identified [40]. The limitations of the current studies in measuring these effects could be assessed by applying different methods in health economics. Discrete choice experiment (DCE), for example, has been proposed as a more reliable method for eliciting women’s preferences for maternity care [64]. DCE assesses and measures the costs associated with consumer preferences for health care by asking pregnant women what they want.

With respect to intrapartum care, while studies show resource inputs and cost ratios for mode of birth to be relatively consistent among countries over time, recent comparison of the costs of childbirth show significant cross-country variation. Factors that have been associated with inter-country cost increases relate specifically to workforce salary rates and provider charges in fee-based health systems [24]. Further, overuse and underuse of birth interventions, for example surgical birth, which may be more prevalent in women who experience high risk pregnancy also demonstrate significant variation and remain subject to multiple influences, including health provider, health system, and funding model [25, 65]. Data from all-risk pregnancies also show that birth by caesarean section (CS) costs substantially more than vaginal birth [66]. International cost ratios for mode of birth validated in Scotland, England, and Australia indicate the incremental equivalent cost ratios as: vaginal birth = 1; instrumental birth = 1.3; caesarean = 2.5 [44]. However, in this review no studies applied or modelled these cost variations for intervention nor linked health outcomes specific to women with pregnancy risk factors in comparative models of maternity care. Despite this, outcomes in some of the studies included in this review show that costly intrapartum interventions, including surgical birth in women with pregnancy risk factors may be safely reduced in intrapartum care for some women who receive continuity of midwifery care thereby also resulting in some cost saving [37–39].

Place of birth is strongly associated with cost [22, 23, 67]. Cost is increased in hospital settings and compounded in facilities with fragmented models of care [68]. However, women with a complex pregnancy currently access many fragmented maternity models and a significant amount of their care in hospitals [11, 31]. The model of maternity care therefore is an important issue when considering cost. The recent introduction of a national maternity care classification system (MaCCs) by the Australian government will enable improved comparison of outcomes and cost between midwifery continuity of care and other maternity models [69, 70] .

In different maternity models and among different provider groups, increased rates of surgical birth, especially caesarean section, and other routine medical practices associated with the cascade of intervention in childbirth increase cost and morbidity [9, 43]. Longer bed stay associated with over intervention for women and their infants results in increased rate and length of hospitalisation, including admission and readmissions, and additional cost in the antenatal, intrapartum, and postpartum periods [31, 69, 71]. The potential savings from improved clinical outcomes generated through midwifery continuity of care across the childbearing continuum should be further evaluated in woman who experience high risk pregnancy and should include the postnatal period [62].

Most published studies have focused on women considered low risk for developing complications and receiving midwifery-led care. Robust evidence from international and Australian studies demonstrates improved cost and clinical outcomes for these women and their babies across a number of key areas, notably physiological vaginal birth [9, 20].

Significantly, midwifery models for low risk women have shown a trend to variable cost saving in health service models where volume is sufficient to achieve efficiency and economies of scale [19, 20]. Savings accrue where caseloads are maintained at an upper threshold of 40 women per midwife per annum [20]. High-volume institutional settings may optimise savings in these models when antenatal hospitalisation rates are kept low, vaginal birth rate is maximised, women and infants undertake early discharge, and receive postnatal follow-up at home or in the community [7, 10, 20, 72, 73]. However, whether these clinical and cost benefits can be extended through greater use of midwifery continuity of care for women who experience pregnancy risk factors require further evaluation. Discrete economic evaluation of midwifery continuity of care in the postnatal period for women with pregnancy risks, as compared to other maternity models including obstetric and standard care was identified as significantly lacking.

In the limited studies examined in this review, diversity in study design and variation in the quality of the results generated often negate reliable comparison of cost results. Where studies include women of mixed pregnancy risk, variation and inconsistency of both study design and the methods applied precluded reliable, comprehensive cost comparisons across the maternity care continuum for woman with pregnancy risk factors. Robust economic evaluations conducted alongside a RCT were considered to have high validity and reliability. None, however, focused exclusively on women with complicated pregnancy. This was in contrast to non-randomised retrospective audit studies [40, 41]. Studies in which a variety of statistical imputation methods or expert opinion or estimates were used to account for missing data created further challenges for reliability in establishing the cost accuracy results of the economic evaluation [41, 74].

Methodological challenges were identified in this review. The first of these was selective risk sampling. Of studies included in this review some used “mixed risk” pregnancy samples that did not stratify clinical results or cost specific to the high-risk sub-set within the sample, thereby limiting generalisability of results. A second challenge included the variables selected for measurement in each study. The variables selected showed significant variation. Accurate measurement of variables depended on the data available and the reliability of the data sources. The data sources of studies included in this review demonstrated wide fluctuation in reliability and quality across different time horizons making comparison of outcomes and cost unreliable.

Inconsistencies that compounded the methodological challenges outlined above also were identified in relation to the various type of economic evaluations of maternity care identified in this review – cost-effectiveness, cost consequences analysis, and economic synthesis. These included the use of varying cost methodology and study assumptions. For example, ‘top-down’ costing approaches that used diagnostic related groups cost weights reflected activity-based funding models [10, 38], as contrasted with ‘bottom-up’ costing that incorporated measurement of specified resource components – for example equipment, consumables, staff salaries, caseload numbers, infrastructure costs [37, 46]. Moreover, sensitivity analysis was included in some of the economic evaluations and not in others. Incomplete or significant amounts of missing data replaced with estimates also called into question the reliability and transferability of cost estimate results. Even synthesis of results from RCTs that applied the most rigorous health economic measures of INB, NMB, NHB and QALYs in the systematic review conducted by Ryan et al [20] estimated projected costs for midwife continuity of care that fluctuated from significant aggregate saving and QALY gains to significant aggregate loss and QALY reductions when assumptions were challenged.

## Conclusion

Robust evaluation and comparative cost performance of alternative models of maternity care is an important consideration in the provision of safe, quality maternity services for women who experience complicated pregnancy. While it is well known that poor outcomes at start to life contribute to long-term chronic disease states that is costly for the health system, optimising clinical effectiveness outcomes and cost efficiency for care of women who experience complex pregnancy requires higher prioritisation. This review found limited evidence to support the cost-effectiveness of midwifery continuity of care for women with complex pregnancy. Further evaluation of cost, resource use and clinical outcomes comparative to other models of maternity care is critical. Further, this review shows that those studies that have attempted to measure these costs demonstrate a range of inconsistencies. The application of inconsistent method undermines valid cost comparison of maternity models in developed countries. This remains an ongoing challenge for policy makers and service providers in implementing system change.

Equitable access to continuity of midwifery care is an important issue for women with pregnancy complication. Evidence on the comparative cost-effectiveness resource use and clinical outcomes delivered through new maternity services is essential to the development of sustainable maternity models. This issue has relevance in an increasing number of settings in Australia and other high resource countries in which services that address healthy start to life are critical to reduce current maternal newborn health inequity, and to meet the needs and expectations of women and their families.

## Abbreviations

AUS: Australia
CI: Confidence interval
CS: Caesarean section
DCE: Discrete choice experiment
DRG: Diagnostic Related Group
INB: Incremental Net Benefit
NHB: Net Health Benefit
NHMRC: National Health Medical Research Council
NHS: National Health Service
NICE: National Institute Clinical Excellence
NMB: Net Monetary Benefit
QALY: Quality Adjusted Life Year
RCT: Randomised controlled trial
UK: United Kingdom
